# The NIRS Analysis Package: Noise Reduction and Statistical Inference

**DOI:** 10.1371/journal.pone.0024322

**Published:** 2011-09-02

**Authors:** Tomer Fekete, Denis Rubin, Joshua M. Carlson, Lilianne R. Mujica-Parodi

**Affiliations:** 1 Department of Biomedical Engineering, State University of New York at Stony Brook, Stony Brook, New York, United States of America; 2 Department of Applied Mathematics and Statistics, State University of New York at Stony Brook, Stony Brook, New York, United States of America; Institute of Psychology, Chinese Academy of Sciences, China

## Abstract

Near infrared spectroscopy (NIRS) is a non-invasive optical imaging technique that can be used to measure cortical hemodynamic responses to specific stimuli or tasks. While analyses of NIRS data are normally adapted from established fMRI techniques, there are nevertheless substantial differences between the two modalities. Here, we investigate the impact of NIRS-specific noise; e.g., systemic (physiological), motion-related artifacts, and serial autocorrelations, upon the validity of statistical inference within the framework of the general linear model. We present a comprehensive framework for noise reduction and statistical inference, which is custom-tailored to the noise characteristics of NIRS. These methods have been implemented in a public domain Matlab toolbox, the NIRS Analysis Package (NAP). Finally, we validate NAP using both simulated and actual data, showing marked improvement in the detection power and reliability of NIRS.

## Introduction

Near infrared spectroscopy (NIRS [1]; for a recent review see [2]), is a non-invasive optical imaging technique that measures changes in oxygenated and deoxygenated hemoglobin concentrations, often in response to changes in neuronal activity. While similar to fMRI's blood-oxygenation-level-dependent (BOLD) outputs for cortical regions, NIRS offers important advantages of low cost, portability, and the ability to extend research to young children and within more ecological environments. Most importantly for adapting dynamical analyses developed for fMRI [3], [4] is the superior temporal resolution, which affords additional information needed for reliable estimation of temporal features of the hemodynamic response for diagnostics. This, however, comes at the expense of limited spatial resolution and restriction to imaging superficial cortical structures. NIRS allows penetration of about one cm into the cortex, due to the light absorption properties of brain tissue, and spatial resolution of about 2 cm, as the source to detector distance must be large enough so that collected photons will be sensitive to tissue absorption [5]–[8].

Because NIRS is still a relatively new modality, it suffers from the lack of a universally accepted framework for data analysis. Given that fMRI, a mature experimental modality, also measures hemodynamic responses, one logical approach has been to adapt well-established methods optimized for fMRI [9] “as is” to NIRS data (e.g. [10]). However, doing so runs some risks if the unique characteristics of the NIRS signal, and even more importantly its noise, do not meet basic preconditions required for valid statistical inference within the framework of the general linear model (GLM).

**Figure 1:**
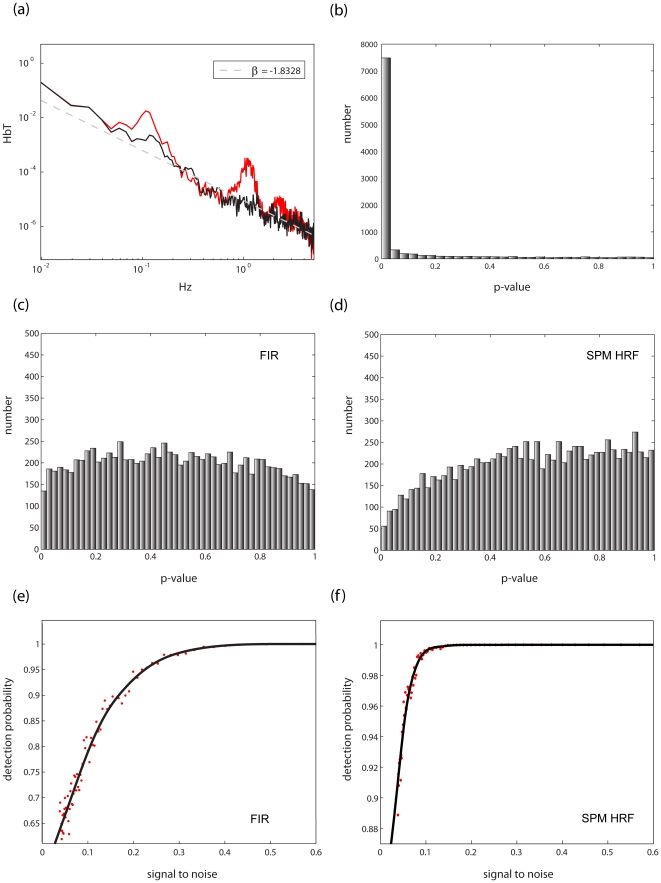
Noise characteristics of NIRS DATA. **(a)**: A representative power spectrum of a NIRS time series captured in the absence of stimulation (rest data in *red*) displayed on a logarithmic scale. The power spectrum follows a power law (exponent ∼ −1.8) tightly, apart from conspicuous deviations caused by systemic artifacts. The spectrum of the same time series after artifact reduction (see Methods section 2.1) is plotted in black. **(b)** Data were simulated data to mimick NIRS rest data (exponent −2). Ten thousand random vectors of this type were generated and analyzed with a GLM representing the design of the task described in Methods section 1, applying the precoloring method [23] to subdue autocorrelation. Next an ANOVA (for the FIR model) or a t-test (for the SPM-HRF model) wass carried out on the resulting coefficients, and the significance (p-value) for each derived. The resulting histogram of p-values is shown **(c)** A similar set of simulated noise was fitted with a FIR model representing the task design, and the FGLS (feasible generalized least squares- i.e. whitening according to a power law fit + precoloring (0.017 Hz)) method described in Methods section 2.3 was applied. The resulting histogram of p-values is very close to the theoretical optimum. **(d)** Simulated noise was fitted with a single basis function – the SPM hemodynamic response function [9] and the FGLS method described in Methods section 2.3 was applied. The resulting histogram of p-values exhibits some slight negative bias. **(e-f)** The same design vector was convolved with the SPM HRF. It then served as a model signal to which simulated noise of varying degrees was added. Next, FGLS was carried out using both the FIR and SPM HRF model. Probability of detection (average p-value) as a function of signal to noise (ratio of RMS (root means squared) squared) for the FIR (e) and SPM HRF model (f) are shown. As can be seen, both models are highly sensitive in the face of noise although, unsurprisingly, given that in the case of the SPM HRF model the model and signal are identical, the SPM HRF model is more sensitive in this case.

In the context of functional imaging of task related activity, error or “noise” is defined as activity uncorrelated with the experimental manipulation; this includes a combination of “spontaneous” hemodynamics (reflecting ongoing brain activity), various sources of physiological activity (such as heart pulsation), and measurement noise. The most striking feature of such resting data is apparent when the power spectra of the data are considered. As can be seen in Fig. 1a, resting data spectra closely follow a power law; that is, when the power spectra are plotted on a logarithmic scale, a linear relation appears to hold between the frequency and its magnitude.

The power spectrum of a time series is the Fourier transform of its autocorrelation function (Wiener–Khinchin theorem); thus, deviation from a flat power spectrum indicates degree of serial correlation. More specifically, data that exhibit strong exponential decay of power will also exhibit high positive autocorrelation in low frequencies. Such autocorrelation is devastating in the context of GLM analysis; while it does not result in bias in the estimates of the parameters of the linear model, it nevertheless leads to underestimation of the noise variance, and therefore to inflation of estimated statistics (such as the *t* statistic, which is inversely proportional to the estimate of the noise variance).

Since the noise correlations generally follow a power law, they can be explicitly modeled as such by calculating their exponent. However, to do so reliably corrections need to be made to suppress significant deviance from the power law at specific frequency bands. These deviances result from systemic physiological artifacts (in this case cardiac pulsations occurring around 1 Hz and blood pressure waves at approximately 0.1 Hz [11] - Fig. 1a). Hence, elimination of systemic artifacts would afford two benefits: not only the ability to reliably estimate the noise autocorrelation structure, but also to increase signal to noise ratio by abolishing excess power in the artifact frequencies.

**Figure 2:**
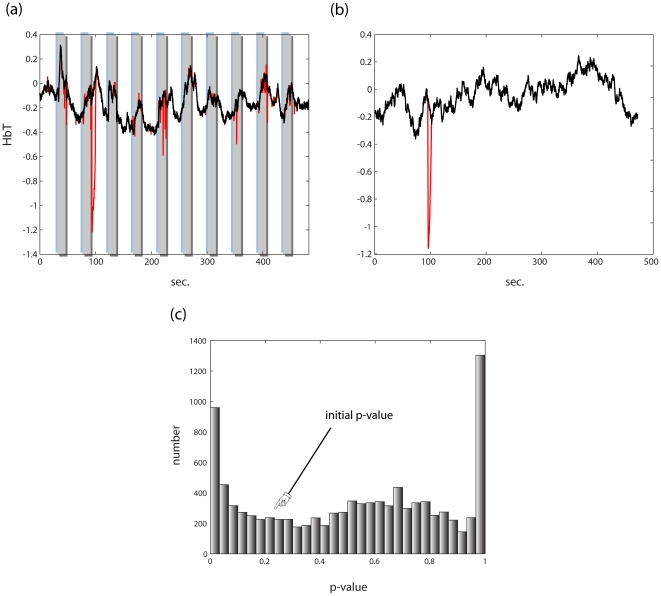
Reduction of motion artifact. **(a)** In *red*, a NIRS time series contaminated by a motion artifact. Superimposed on it in *black* is the time series resulting from artifact cancellation. **(b)** In *red*, a simulated NIRS time series (phase randomized noise, exponent −2) to which a simulated motion artifact is added, matched to the one in (a) by amplitude and duration. In *black* is the time series resulting from artifact cancellation. **(c)** The time series in (b) was analyzed using the SPM hemodynamic response model and the feasible generalized least squares FGLS (feasible generalized least squares – i.e. whitening according to a power law fit + precoloring; 0.017 Hz) method described in Methods section 2.3, resulting in a p-value of 0.227. A set of 10000 artificial motion artifacts varying only in time of onset was generated and added to this time series and analyzed similarly. The resulting histogram of p-values demonstrates that motion artifact negation is imperative for valid statistical analysis. After motion artifact cancellation the correlation of these data to the original time series was 0.996±0.004, and the resulting p-value was 0.237±0.068 (reflecting the slight negative bias shown in 1(c)).

A second critical impediment to reliable statistical inference in NIRS is the effect of motion artifacts on NIRS data, which are caused mostly by head movement [12]–[16]. NIRS imaging requires optodes to be placed against participants' scalps. As the optodes press against a participant's skin, gross head movements compromise the contact between the optodes and skin, in effect changing the refractory constant and the source-to-detector distance seen by the NIR beam (Fig. 2a). One of the consequences of such artifacts is a substantial statistical bias towards false positives [2], [15], which must be subdued to achieve efficient statistical inference.

Below we show how the noise inherent to NIRS can be modeled and controlled for, thus enabling to achieve near optimal statistical inference. The methods we describe have been implemented in our NIRS analysis package (NAP; http://lsec.neuropraxia.webfactional.com/Software_and_Instrumentation.html), a public domain Matlab (Mathworks, Natick MA) toolbox.

## Methods

### 1. Experimental materials and methods

#### Participants

We recruited 12 adults (three females), ages 23-39 to participate in the experiment. All subjects were healthy and free of neurological or cardiovascular illness. This study was approved by the Institutional Review Board of Stony Brook University School of Medicine; all participants provided informed written consent.

#### Experimental procedure

Participants were presented with a full-screen checkerboard stimulus flickering at 8 Hz [17], interspersed with an equiluminant screen, in a 15/30 sec block design (10 repetitions).

#### Data acquisition

NIRS data were collected using the ETG 4000 (Hitachi,Tokyo) with 33 probes (52 channels) placed occipitally, and sampled at 10 Hz. The ETG 4000 employs two lasers with wavelengths of 695 nm and 830 nm, both with optical intensity of 2mW. Optodes were secured to the skull using a custom-made cap and participants' heads were placed in a custom-made head holder. Optode locations and skull markers were measured using a Polhemus ISOTRAK II magnetic tracker (Inition, London) controlled by the ETG 4000. The 10-20 markers [18] measured were: Nz (nasion) Iz (inion) AR (right ear) AL (left ear) and Cz (midpoint of the crown of the head).

### 2. Data analysis

Data were analyzed using the NAP. First, data were cleaned from movement artifacts and systemic noise (heart beat cancelation followed sequentially by movements, breathing and blood pressure artifact cancelation). Next, data were analyzed using the feasible generalized least squares (FGLS) scheme described below (i.e., precoloring with a cutoff of 1.7 Hz and/or whitening with a power law derived noise covariance matrix) employing both an SPM HRF model and an FIR (finite impulse response) model (spanning 20 sec.). To save computation time, data were temporally binned by a factor of three (reducing the FIR coefficients to 67) before carrying out inference. Single subject optode locations were registered to the MNI (Montreal Neurological Institute) template space, channel locations computed using the method described in [19]. Group analyses were carried out using the method described in Beckmann et al. ([20], see appendix S3).

### 2.1. Correction of periodic physiological artifacts

**Figure 3:**
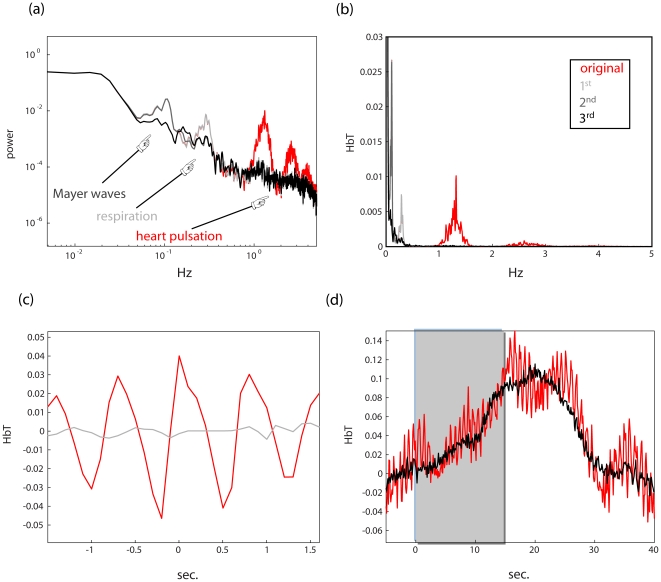
A recursive algorithm for systemic artifact reduction. **(a)** A power spectrum of an activated NIRS channel (*red*) on a logarithmic scale. The spectrum exhibits excess energy in several frequency bands associated with periodic systemic artifacts: heart pulsations (1–2 Hz in adults), respiration (∼0.4 Hz in adults), and blood pressure (Mayer) waves (∼0.1 Hz). Superimposed on this are the spectra resulting from each successive application of the algorithm described in Methods section 2.1: in *light gray* after heart beat is removed (i.e. using [0.6 2] Hz), in *dark gray* after the second sweep, for respiratory artifacts (i.e. using [0.15 0.4] Hz), and finally in *black* after using [0.05 0.2] Hz for blood pressure waves. **(b)** The same plot in linear scale. **(c)** After the first sweep the NIRS time series was averaged around the detected heart beat onsets. As can be seen in *gray*, the artifact is all but gone as compared to averaging the original time series in the same fashion. **(d)** The above time series was averaged around stimulus onset (see Methods section 2.1 for details) before (*red*) and after (*black*) the procedure. As shown, the resulting response doesn't exhibit conspicuous oscillatory components.

While NIRS measurements capture aspects of the underlying hemodynamics in the imaged tissue, the resulting time series reflect not only functional changes but underlying physiological processes that are not directly coupled to neuronal activity. Among the primary influences are cardiac pulsation, as well as respiratory and blood pressure waves. Accordingly, NIRS time series usually exhibit conspicuous periodic artifacts arising from these oscillatory systemic processes (Fig. 3a). However, as such artifacts are quite regular in time and exhibit moderate variation from a stereotypical form associated with each such process, NIRS data provide sufficient information to model these processes and thereby eliminate them.

In the neuroimaging literature, two methods have been suggested to explicitly model cardiac pulsations: an event-triggered averaging method utilizing independent cardiac measurements (i.e., [21]) and a stand-alone method employing time-warping averaging (i.e., [22]). In what follows, we present a standalone method for periodic physiological artifact removal, which synthesizes, extends, and generalizes the aforementioned methods. The proposed method capitalizes on the fact that systemic artifacts are contained in relatively restricted bandwidths of measured signals. Therefore, the same algorithm can be applied recursively by moving from higher frequency artifacts to lower frequency ones (e.g., beginning with correcting cardiac artifacts and then proceeding to correct respiratory artifacts).

For each systemic artifact, beginning with high frequency events, the following steps are proposed:

- Average data from different channels to enhance artifact: As systemic artifacts result from a unitary underlying cause (e.g., [21]), averaging across channels reduces both noise and spontaneous hemodynamic components arising from local spontaneous neuronal activity, enhancing the trace of the systemic process (see Fig. S1). It is more effective to discard noisy channels before averaging, to facilitate artifact detection. A simple way to achieve this is selecting channels with low variance. For example, the channel variances can be z-transformed and only the channels with a variance lower than one standard deviation averaged.
- Band-pass filter the resulting time series to the relevant bandwidth: For example, in the case of adult data: relevant bandwidths are [0.6–2.0] Hz for cardiac pulsations, [0.15–0.4] Hz for respiratory artifacts, and [0.05–0.2] Hz for blood pressure waves.
- Detect systemic events by identifying the local minima and maxima of the signal: The most straightforward way of doing this is to compare the signs of the derivative function to the left and right of each point in a time series. A local maximum is characterized by the derivative being positive in a neighborhood to the left of the point, followed by a change in sign to the right, and vice versa for local minima. The sign of the derivative to the left of 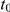 can be approximated by 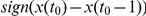 and similarly for the sign on the right.
- Identify time-invariant signature waveforms (i.e., “time-warping”) associated with each type of artifact by from local extrema: We aim here to provide a time-invariant “signature” waveform associated with each type of systemic artifact (e.g. a characteristic heart pulsation artifact). First, segment data between local minima and maxima. Secondly, divide segments into two groups: low to high phases (segments between consecutive minima and maxima) and high to low segments. Compute the average length (number of samples) of segments in each group. Resample each of the low to high segments (using linear interpolation) so they encompass the same number of samples (the average length). Thus if a time series of length *k* is to be resampled to comprise *l* timepoints, if we denote the original time series *x* and the resampled one 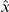, then it is assumed that 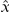
(1) * =  x*(1), 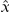
(*l*) * =  x*(*k*) and in general that 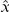
(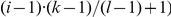) * =  x*(*i*). Finally, average all the resampled low to high segments. Repeat the same procedure for the high to low phases. Combine the average low to high segment with the average high to low segment to form one time series: the time-warped wave form. If necessary, the low to high and high to low segment values are adjusted so that the ends will coincide (i.e. that the high to low template will end with the same value of the first value of the low to high template).
- Resample this template according to the detected events: “stretch” and “contract” it to conform to detected local minima and maxima using linear interpolation. This results in an estimate of the artifact time series – e.g. a time series containing pulsations occurring and the same time point as heart pulsations detected by the method above.
- Remove the estimate of the artifact from the original time series: For each time series, the estimate of the artifact in that channel is subtracted from the original series.
- Average the resulting time series around event onsets (i.e. local maxima) to obtain the residual.
- Remove the residual from the time series: Replicate and realign the resulting template according to original local maxima times and subtract.

The reason for utilizing both time-warped averaging as well as event-triggered averaging is twofold. First, while time-triggered averaging is by definition unbiased in the window around the event onset (the length of which is the minimal event-to-event time-difference) it does not sufficiently reduce the average magnitude of the events preceding and following the trigger. Second, time-warped averaging is slightly biased within the event-onset window; however, not outside of it. Therefore, the combination of the two is superior to the application of each method on its own, as systematic biases are thereby avoided.

For low frequency artifacts (i.e. respiratory and blood pressure waves) it might be more prudent to average the band passed signal, rather than the original, since the fact that these events are slow means that they are relatively infrequent in the time series. Therefore, low frequency signal modulations (e.g. response to stimulation), which contain the lion's share of energy of the signal might not average out sufficiently. Although normally deoxyhemoglobin time series do not exhibit cardiac artifacts, at times residual artifacts are nevertheless present. In such cases the corresponding oxy-hemoglobin data can be used for artifact detection and the remaining steps carried out as described.

### 2.2. Motion artifact correction

Motion artifacts are manifested in NIRS data as brief signal inflections of one to two orders of magnitude larger in amplitude than the hemodynamic signal. Several methods for motion artifact detection and cancellation have been proposed in the NIRS literature [12]–[15]. Unlike some of the global methods that modify the entire time series (which would again adversely affect dynamical analyses), we describe a more targeted approach below that explicitly detects and approximates motion artifacts, rectifying the signal only in the contaminated segments. The proposed method includes two phases: detection of artifacts, followed by their reconstruction. We found that for this purpose it is useful to classify artifacts into two categories: *spikes*, which are near-instantaneous signal inflections, and *ripples,* which are prolonged events sometimes spanning up to several seconds.

As explicit detection of events requires a threshold, data are first preprocessed in the following manner to enable standardization and therefore automation of threshold selection:

- Highpass filter the time series. We applied a 4^th^ order Butterworth filter with a cutoff frequency of 0.01 Hz.
- Apply a z-transform to the time series. (i.e. remove the time series' mean and divide by its standard deviation)

To detect and cancel ripples we suggest the following procedure:

- Detect ripple apexes: Segments of the data that exceeded a fixed threshold (e.g., ± four standard deviations) are identified according to the magnitude of their integral (e.g., at least 10 consecutive data points have to exceed the threshold). In each contaminated segment the maximum of the absolute value of the segment was found. The resulting set of points mark the apexes of the movement artifacts in the signal.
- Detect ripple onset and offset: First, define a search window around each side of each ripple apex. Initially, set the window size to a duration that is sufficient to encompass typical artifacts (∼10 seconds). Next, find within that window the global opposite extremum point (maximum if the apex is negative and vice versa). Set the window to encompass several time points (e.g. 10) to the side of the extremum point (left if detecting onset and vice verse). Then, segment the data within the resulting window into three segments on each side, using a greedy top down/bottom up algorithm (see Appendix S1). This allows the detection of the change points marking the onset and offset of artifacts (i.e., it is the end of the first segment for onsets and the end of the second segment for the offsets).
- Approximate the artifact using piecewise continuous low order polynomials: approximate each phase of the ripple with a 3^rd^ degree polynomial and subtract the estimate from the original time series. This allows preserving the high frequency content of the original time series (thus preserving the power law structure of the signals' power spectrum). This is important for later estimation of the noise autocorrelation when inference is carried out (see Methods section 2.3)

To detect and cancel spikes we suggest the following procedure:

- Find the local extrema of the time series (local minima and maxima using the procedure described above)
- z-transform the magnitude of the peak to valley and valley to peak changes.
- Find consecutive inflections that exceed a threshold (e.g. a peak to valley segment followed by a valley to peak segment both of a magnitude exceeding 2.5 standard deviations).
- Interpolate data in the implicated time points (consecutive segments): As spike artifacts comprise a scant number of samples (typically 3–4 time points at 10 Hz), more complex reconstruction methods are uncalled for. Accordingly spike detection is appropriate only for high sampling rates (e.g. 10 Hz, the standard sampling rate of many commercially available NIRS systems)

Conceptually, it might seem reasonable to first treat movement artifacts before proceeding to tackle systemic ones such as cardiac pulsations. However, since the algorithm described above rests upon simplification of the NIRS time series (i.e., segmentation into non-overlapping line segments), we have found it more effectual to first clean the signal from the heartbeat pulsations before proceeding to detect movement artifacts.

### 2.3. Inference of activation

In this study we applied two GLMs, the SPM GLM [9], and a FIR model comprising 20 seconds (in 10 Hz 200 basis vectors).

To combat serial correlation we used either feasible generalized least square analysis (FGLS), prewhitening [23], or both.

In FGLS, ordinary least squares (OLS) is carried out first, followed by estimation of the noise covariance utilizing the residuals under the assumption that they conform to a presupposed structure. In the case of NIRS data, this is sound practice because the structure of the noise covariance in a given cortical locus is known up to a single parameter, namely the exponent of the power spectrum [24].

The procedure of estimating the noise covariance matrix includes the following:

- Carry out OLS regression (see Appendix S2) and compute the residuals 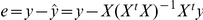

- Compute the power spectrum of the residuals 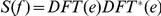 (* denotes the complex conjugate).
- Regress the power spectrum to a line after transferring to log units: regress log(f) to log(S(f)) to find the slope 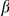

- Compute the autocorrelation function: i.e., 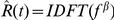

- Build the covariance matrix: Note that the covariance matrix is simply another way of arranging the autocorrelation function, namely: 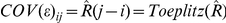

Once the covariance matrix is found, which we will denote *V,* it can be inverted and used for GLM analysis.
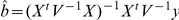


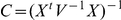


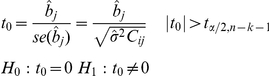


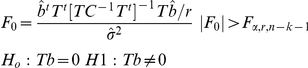


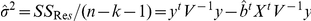
Precoloring was implemented by high-pass filtering (via a 4^th^ order Butterworth filter) the data and design matrix (sparing the initial column to keep the regression scheme intact). If only precoloring was applied, it was followed by OLS (see Appendix S2 for a detailed account). If both precoloring and FGLS were combined, after precoloring the exponent of the residual power spectrum was estimated outside the cutoff frequency. Next, the resulting spectrum was multiplied by the filtering kernel, after which whitening was carried out as above using this modified spectrum.

### 3. NIRS Analysis Package (NAP), a publicly available Matlab suite for NIRS analysis

The methods described above have been implemented in our NIRS Analysis Package (NAP) a Matlab (Mathworks, Natick MA) toolbox, which can be downloaded at http://lsec.neuropraxia.webfactional.com/Software_and_Instrumentation.html). NAP enables any combination of cardiac, respiratory, blood pressure, and motion artifact cancellation. While NAP is set to default parameters that we have found to be most effective, motion artifact thresholds can be user specified. Similarly, users can select several predefined settings determining the frequency bands of systemic artifacts according to age (adults, children of ages 5–12, and toddlers of ages 2–5) as well as a custom option.

The toolbox enables researchers to apply either the standard SPM HRF model (with or without derivatives), or an FIR model to single subject data. Either whitening (according to a power law fit), precoloring, or both can be specified to offset serial correlations in the data. Apart from the de-noising methods described here and single-subject FGLS analysis, NAP also includes group analysis capabilities, using either hierarchical GLM analysis (Beckmann, 2003) or the SPM summary statistic method (Friston, 1999).

Finally, NAP enables simulation of hemodynamic responses to arbitrary paradigms, and standalone registration of optode and channel locations to the MNI space (the Montreal Neurological Institute template) by using magnetic measurements of the optode position according to the method described in [19].

NAP provides visualization of the results of GLM analyses as SPMs overlaid on the cortical surface, both at the individual and group level, as well as marking channel and optode loci. Also, NAP includes a channel data plotting module. The channel plotter allows plotting data with or without additional filtering, as well as plotting data power spectra and event-triggered averages.

## Results

### 1. Systemic artifact cancelation

The results of applying NAP's systemic artifact cancelation to NIRS data are shown in Fig. 3, in which data resulting from visual stimulation with a flickering checkerboard stimulus (see Methods section 1) were cleaned of heart pulsations, respiratory waves and finally blood pressure waves in succession. In Fig. 3a–b, the power spectra of the successive stages are shown, demonstrating that each iteration of the algorithm abolishes the excess power in the frequency band associated with each systemic artifact in turn. In Fig. 3c, we show the result of averaging the time series around detected heart pulsations before and after heartbeat cleaning, illustrating the effectiveness of the method. Finally, Fig. 3d shows the average response to a visual stimulus for this channel. The original (red) exhibits noticeable cardiac pulsations as well as respiratory waves. They are all but gone from the corrected signal, but without the loss in high frequency power that would have resulted from ordinary filtering (for a discussion of the effectiveness of filtering in inference see Results section 3 and Friston et al. [23] and Smith et al. [25]), which would negate the advantage of temporal resolution for dynamical analyses obtained from NIRS.

On average systemic artifact cancellation resulted in an increase in signal to noise by a factor of 4.33.

### 2. Motion artifact cancellation

To quantify the impact of motion artifacts on inference, we simulated typical NIRS noise (i.e. colored, or 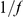 noise), then proceeded to carry out statistical inference on each simulated artifact vector using a GLM (corrected for autocorrelation as described in Methods section 2.3) representing a 15 sec stimulus alternating with a 30 sec rest block design with TR = 0.1 sec using an SPM-like HRF regressor [9]. We chose a specific instance of noise whose significance under this model was *p* = 0.227, and added to it a simulacrum of the observed artifacts both in magnitude and duration at random times (Fig. 2b). We then proceeded to carry out the same GLM analysis on 10000 noise vectors of this type (i.e. each containing the original time series to which a simulated artifact was added at a random time), and then calculated the distribution of resultant *p*-values. As can be seen (Fig. 2c), artifacts of this magnitude completely mask the underlying signal, thereby rendering subsequent GLM analysis invalid, and therefore must be corrected for reliable statistical inference.

We applied the method in Methods section 2.2 to the simulated data, and analyzed them with the same model, which resulted in p = 0.227 for the original noise vector. The resulting correlation coefficient between the rectified time series and the original time series was 0.996 on average (s.d.  =  0.00043). The correction, as shown in Fig. 2, enabled reasonable subsequent inference; the resulting p-value was .237±.068 on average.

An example of the above de-noising procedure on a NIRS time series is shown in Fig. 2a. It illustrates that artifacts can be abolished without compromising functional changes, although these involve abrupt changes that would be picked up by simply applying a change point methodology. Rather, the proposed method is custom-tailored to the statistical nature of the observed motion artifacts and not simply to massive violation of stationarity (as in a functional response). For a comparison to two other publically available methods see Fig. S2.

### 3. Reliable inference of activation

To compare various schemes of inference, we simulated NIRS resting data, or noise, by generating colored noise sampled at 10 Hz with exponents (slopes in the logarithmic representation of the power spectra) in the range found in actual data. This was achieved by phase randomization of the Fourier sinusoidal components such that their combined magnitudes conform to a desired power law [26]. Next, these data were fitted with a GLM representing a 15–30 sec. block design (mimicking the design employed in our study; see Methods section 1). Next an ANOVA (for the FIR model) or a t-test (for the SPM-HRF model) is carried out on the resulting coefficients, and the significance (p-value) for each fit derived. Colored noise vectors are directly sampled from the null hypothesis in the scenario of NIRS GLM analysis; therefore, the resulting histogram of *p*-values should be flat, as is the case when OLS regression analysis is carried out on white noise. As can be seen in Fig. 1b, however, analyses of uncorrected data result in severe bias.

In theory, the optimal strategy for dealing with biases resulting from such autocorrelated noise is through whitening (see Methods section 2.3). However as noted by Friston et al. [23], while whitening is the most efficient method in theory, its use can backfire in the case of model misspecification. They suggest that this risk can be minimized by *precoloring*, multiplying both sides of the regression problem by a linear filtering matrix (i.e., filtering both data and the design matrix), rather than by a whitening matrix derived from the covariance estimate. Unlike a recent recommendation of precoloring as a method of choice in NIRS analysis [i.e., 10], this approach proved to be ineffective on simulated NIRS noise (see Fig. 1b). However, as noted by Smith et al. [25], precoloring (especially with a high-pass filtering matrix) can be combined with other whitening schemes, thus, possibly enjoying the best of both worlds. As seen in Fig. 1(c–f), this approach works quite well on the simulated noise and as we will show now it is indeed far superior to both whitening and precoloring on their own when applied to actual data. Additionally, applying motion artifact and systemic artifact cancelation to such noise did not compromise inference (Smirnov Kolmogorov test on the p-value distributions, p>.8 for SPM-HRF model, p>.6 for the FIR model, n = 1000).

**Figure 4:**
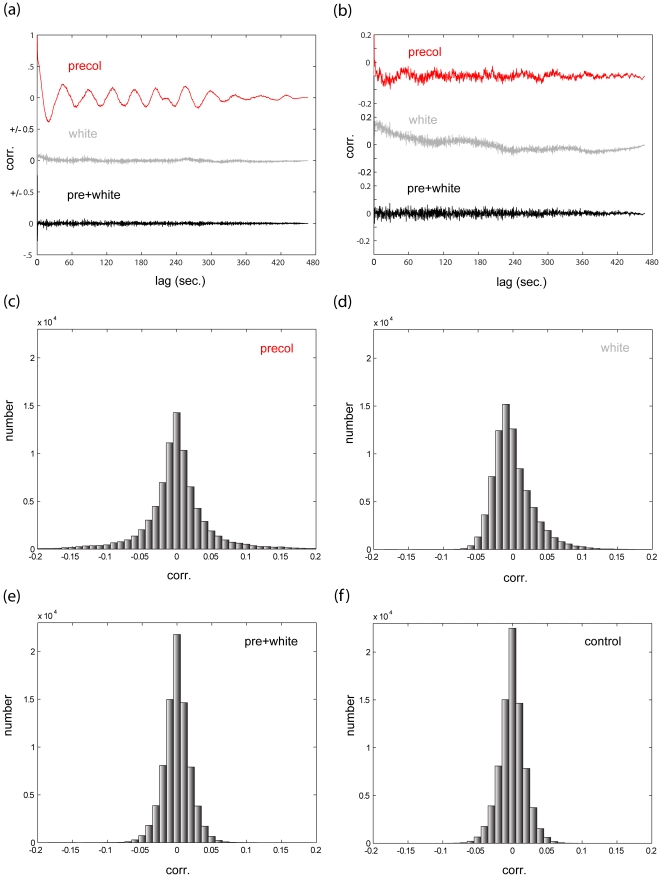
residual analysis. **(a)** Results of residual analysis (channel 23, participant 5). In *red*: the empirical autocorrelation of the residuals after fitting the time series with an SPM HRF model with precoloring. In *gray*: the results of similar analysis, this time using whitening (see Methods section 2.3). In *black*: the results using both. In this example, while whitening substantially outperforms precoloring, it is nevertheless improved upon by the combined approach. **(b)** Results of residual analysis (channel 46, participant 5). Same notation as (a). In this example, precoloring actually outperforms whitening, but again the combined approach is superior to both. **(c**–**f)** A histogram of all residual autocorrelation values (lag>0) for this participant using (c) precoloring (d) whitening (e) both. (f) histogram of all residual autocorrelation values (lag>0) resulting from applying the same design matrix with OLS to white noise. As shown, the combined the FGLS approach we propose is very close to the theoretical limit of efficiency.

To check the robustness of the proposed FGLS scheme, we carried out residual analysis after applying precoloring alone, applying whitening alone, and finally applying both. Fig. 4a–b shows exemplary residual autocorrelation functions; while both whitening and precoloring reduce the autocorrelation in the residuals, only the combination of both achieves satisfactory results. The entire distribution of the residual autocorrelation coefficients for this participant is shown in Fig. 4c. As a comparison we applied the same design matrix to white noise using OLS (Fig. 4d). Clearly, the combined FGLS scheme is superior to both precoloring and whitening, and is nearly as efficient as the optimal scheme in the uncorrelated scenario.

Contrary to our expectation, the FIR model was not effective in resolving the underlying hemodynamic function; only in a small fraction of the activated channels across participants did the impulse response exhibit definite structure.

**Figure 5:**
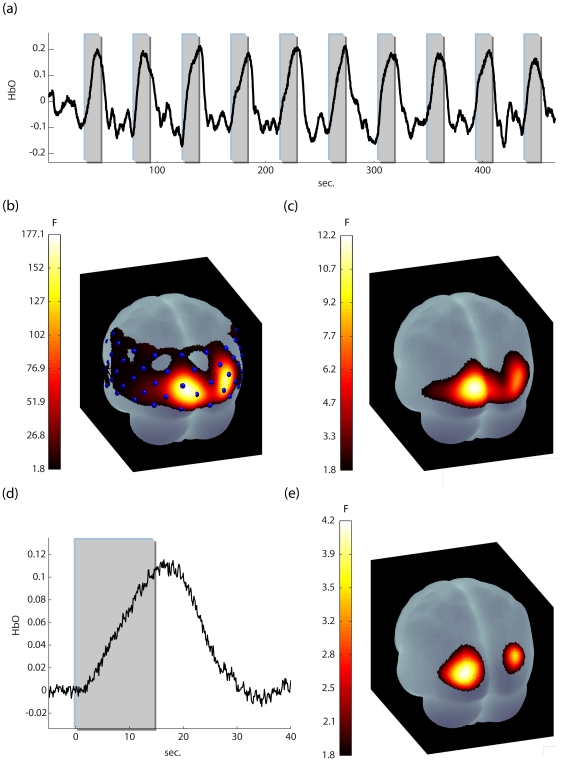
The results of the visual task. **(a)** : a representative time series (Participant 8, channel 26). Data were band-passed filtered for purposes of presentation **(b)** the activation map (oxyhemoglobin data) for this participant, using a FIR model with precoloring (cutoff 0.017 Hz). 46 out of 52 channels are activated for p<0.01 (corrected for channel number). **(c)** the activation using a FIR model with whitening+precoloring (cutoff 0.017 Hz) for p<0.01 (corrected for channel number). Now activation is restricted to the visual areas. **(d)** the average activation for all participants (channel 26, n = 12). Note, that no residual oscillatory artifacts are present. **(e)** the activation map for all participants for the FIR model p<0.01 (corrected for channel number). Group analyses were carried out using the method described in Beckmann et al. ([20], see appendix S3).

A representative response evoked by the visual stimulation is shown in Fig. 5a. We show activation maps for the same participant whose data underwent both the analysis suggested in Ye et al. [10]- FIR-GLM augmented with precoloring, cutoff 0.17 Hz (Fig. 5b) - and analyses following the methods proposed above (Fig. 5c). It is important to note that conventional analyses lead to a great inflation in the estimated *p*-values: 46 out of 52 channels turn out to be significant after Bonferroni correction for multiple comparisons at a threshold of p<0.01. In contrast, the activation map resulting from using the abovementioned FGLS scheme is well contained within the visual cortices at the same threshold.

The average group response (oxyhemoglobin) is shown in Fig. 5d, and although no filtering was applied to the data, there remain no residual systemic effects. The group activation map for the FIR model is displayed in Fig. 5e (p<0.01 corrected). Again activation is restricted to the visual cortices, as appropriate.

## Discussion

### 1. Limitations and future work

Contrary to our expectation, although the NIRS sampling rate is over an order of magnitude faster than standard fMRI, we were unable to resolve the hemodynamic response function using a FIR GLM. Although this might result in part from utilizing a block design, one possibility is that it results from the inherent NIRS signal to noise ratio. If so, it might be appropriate to incorporate in NAP, basis sets that lie somewhere in between the highly constrained SPM model, and the uttermost flexibility of FIR models – e.g. [27]. Future NIRS studies, using event-related designs, will be able to address this issue.

Our first version of NAP is geared toward inference of activation in a classical block or event-related design, since these types of designs still represent the vast majority of studies currently conducted. However in recent years there has been a growing tendency to analyze imaging data either in task-free scenarios or employing nonlinear analysis e.g. [28], connectivity [29] and complexity [3], [4] analyses. In line with this direction, future versions of NAP will incorporate connectivity and complexity analysis tools, which will extend NAPs flexibility in adapting to a wider variety of experimental protocols.

### 2. Conclusion

We have described a complete analysis scheme for NIRS functional data that comprises three major steps:

- Elimination of systemic artifacts, thus increasing signal to noise and enhancing the ability to estimate the structure of the noise covariance
- Elimination of motion artifacts, without which statistical inference is not valid.
- Feasible generalized least squares inference by combining whitening of the data, using the covariance structure resulting from fitting a power law to the residuals of OLS regression, and precoloring (i.e. highpass filtering the data as well as the design matrix [23], [25]).

Applying the described procedure both to simulated data as well as visual stimulation data demonstrates that near optimal inference can be achieved.

These methods have been implemented in NAP (http://lsec.neuropraxia.webfactional.com/Software_and_Instrumentation.html) a public domain Matlab toolbox for NIRS analysis, visualization and anatomical registration. It is therefore our hope that it will serve the community of NIRS researchers and become a platform for additional refinement and improvement of customized NIRS analysis methods.

## Supporting Information

Figure S1 **Global synchrony of systemic artifacts.** Heart pulsations taken from the four corner channels in a 52 channel array support the assumption of synchrony for systemic artifacts. Top: Oxy data from the flickering checkerboard data. Bottom: data collected during viewing of a movie – frontal optode positioning. Pulsations seem to be in perfect sync in the superficial cortical layers seen by NIRS.
(TIF)

Figure S2 **Comparison of NAP to other publically available motion artifact reduction methods.** *Red* – original time series, *black* – modified time series *(a-c)* analysis of the HbO time series originating from the same channel as fig. 2. *(a)* With artifact reduction using NAP, artifacts are reduced without compromising functionally related signal changes *(b)* Artifact reduction using HOMER (i.e. using the method of [16]). To achieve substantial noise reduction it was necessary to discard 10 principal components; however, use of 11 components nearly abolished the functional signal *(c)* Artifact reduction using ([15]; http://www.alivelearn.net/nirs/CBSI.m). Spikes in the data are removed. However this method comes at the expense of losing information in the deoxy signal (i.e. the end result is a modified oxy signal, precluding analysis of both the deoxy and total signals). Further still the global changes in the time series can result in loss of information about various signal features. *(d-e)* Analysis of the time series of fig. 2e, with artifact reduction using HOMER. As can be seen, in this case removal of 10 principal components does not eliminate the artifact, yet compromises the functional signal and even introduces additional spikes. This points at a major weakness of PCA based denoising methods, which is the inability to predefine satisfactory component selection criteria; the number of components necessary to effectuate meaningful change in a time series varies greatly. Moreover, criteria suggested in the literature for selection of components are usually similarity to the task design (e.g., [30]), which in the case of subsequent inference can substantially bias the results. This of course is also true of selection by visual inspection. Aside from having clear cut statistical criteria for theresholding, the NAP movement reduction has the advantage of being local, and hence applicable to wider scenarios than functional imaging, e.g. analysis of resting state data and connectivity.
(TIF)

Appendix S1 **Top-Down/Bottom-Up Segmentation.** Description of the segmentation algorithm derived from [31] incorporated into NAP's motion artifact detection.
(DOC)

Appendix S2 **Single subject GLM analysis with FGLS.** A detailed account of application of whitening using a power law covariance structure to offset autocorrelated errors in single subject inference of activation using a GLM.
(DOC)

Appendix S3 **Group analysis using hierarchical GLMs with FGLS.** A detailed account of group analysis applying a hierarchical GLM incorporating the single subject estimated error covariances to achieve optimal inference (through whitening). Method derived from [20].
(DOC)
