# Supplementary figures and images for: The NIRS Analysis Package: Noise Reduction and Statistical Inference
Source: PLoS One. 2011 Sep 2;6(9):e24322. doi: 10.1371/journal.pone.0024322 (PMC3166314; doi:10.1371/journal.pone.0024322)

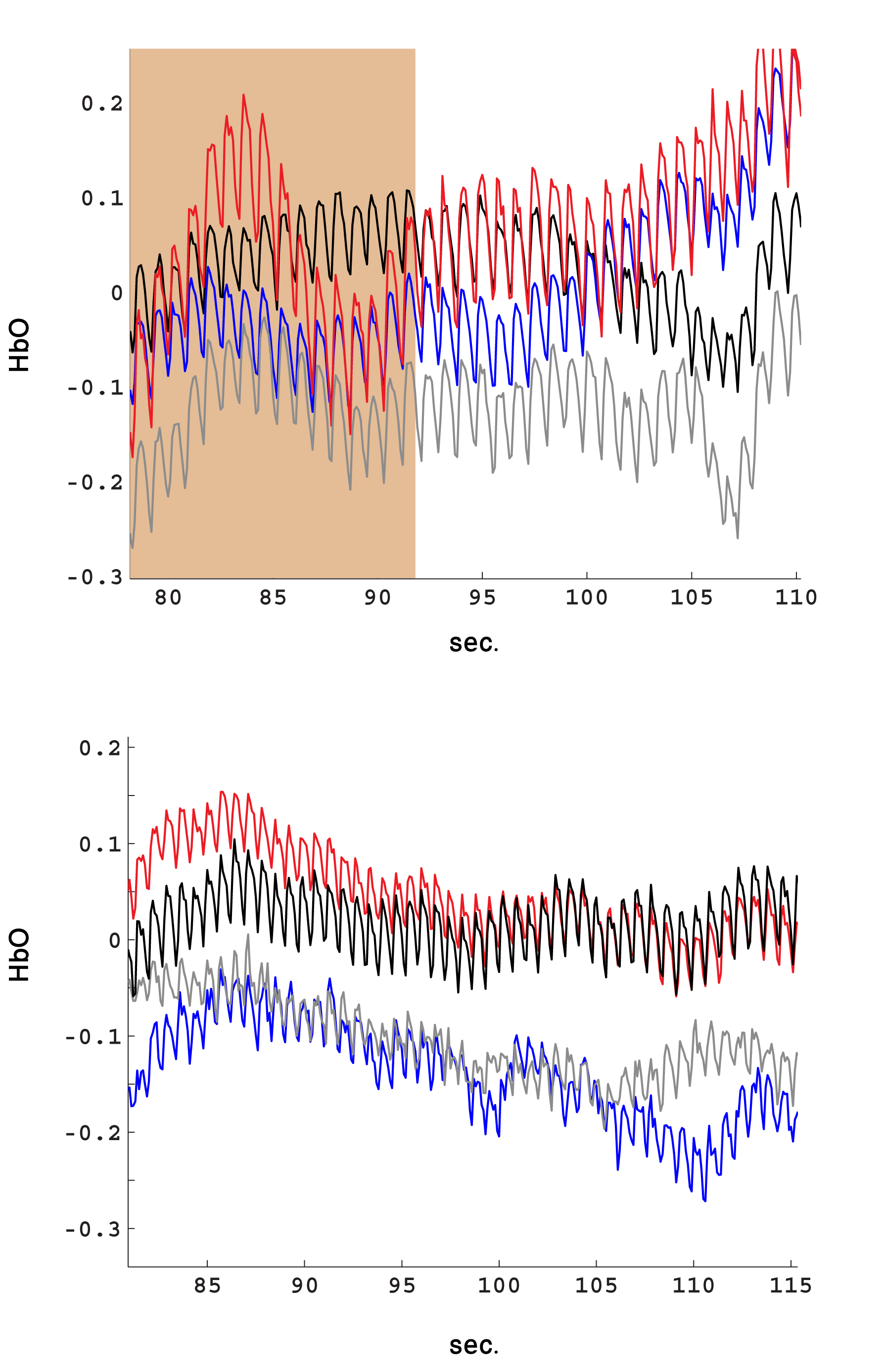

Supplement: Figure S1 — Global synchrony of systemic artifacts. Heart pulsations taken from the four corner channels in a 52 channel array support the assumption of synchrony for systemic artifacts. Top: Oxy data from the flickering checkerboard data. Bottom: data collected during viewing of a movie – frontal optode positioning. Pulsations seem to be in perfect sync in the superficial cortical layers seen by NIRS. (TIF) [file pone.0024322.s001.tif]

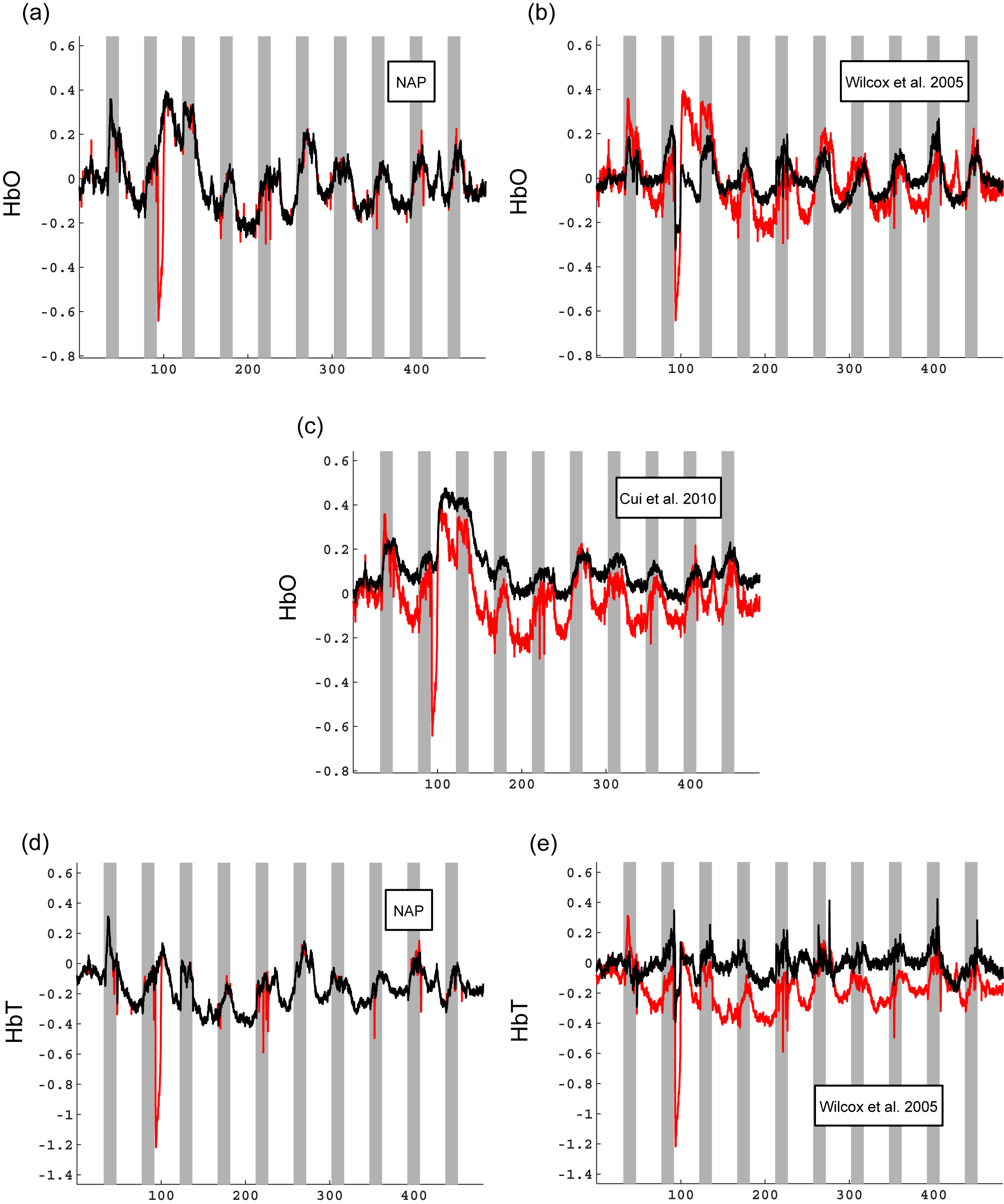

Supplement: Figure S2 — Comparison of NAP to other publically available motion artifact reduction methods. Red – original time series, black – modified time series (a-c) analysis of the HbO time series originating from the same channel as fig. 2. (a) With artifact reduction using NAP, artifacts are reduced without compromising functionally related signal changes (b) Artifact reduction using HOMER (i.e. using the method of [16]). To achieve substantial noise reduction it was necessary to discard 10 principal components; however, use of 11 components nearly abolished the functional signal (c) Artifact reduction using ([15]; http://www.alivelearn.net/nirs/CBSI.m). Spikes in the data are removed. However this method comes at the expense of losing information in the deoxy signal (i.e. the end result is a modified oxy signal, precluding analysis of both the deoxy and total signals). Further still the global changes in the time series can result in loss of information about various signal features. (d-e) Analysis of the time series of fig. 2e, with artifact reduction using HOMER. As can be seen, in this case removal of 10 principal components does not eliminate the artifact, yet compromises the functional signal and even introduces additional spikes. This points at a major weakness of PCA based denoising methods, which is the inability to predefine satisfactory component selection criteria; the number of components necessary to effectuate meaningful change in a time series varies greatly. Moreover, criteria suggested in the literature for selection of components are usually similarity to the task design (e.g., [30]), which in the case of subsequent inference can substantially bias the results. This of course is also true of selection by visual inspection. Aside from having clear cut statistical criteria for theresholding, the NAP movement reduction has the advantage of being local, and hence applicable to wider scenarios than functional imaging, e.g. analysis of resting state data and connectivit [file pone.0024322.s002.tif]
