# Supplementary material for: The NIRS Analysis Package: Noise Reduction and Statistical Inference
Source: PLoS One. 2011 Sep 2;6(9):e24322. doi: 10.1371/journal.pone.0024322 (PMC3166314; doi:10.1371/journal.pone.0024322)
Supplement: Appendix S1 — Top-Down/Bottom-Up Segmentation. Description of the segmentation algorithm derived from [31] incorporated into NAP's motion artifact detection. (DOC) [file pone.0024322.s003.doc]

## Appendix S1: Top-Down/Bottom-Up Segmentation.

The goal of this algorithm is to segment a time series into a designated amount of segments . TopDown segmentation is recursive: it begins by seeking the partition of a time series into two segments that yields the minimal sum of squares. All possible partitions are tried, points in each group are fitted with a line, and the squared errors summed. This procedure propagates until a criterion is reached – in our case two iterations to produce a partition into 4 segments. Next, two consecutive segments need to be merged to yield a three way partition. This is done bottom up by simply choosing the merger that induces the minimal total sum of squares from fitting each of the three resulting segments with a line.
