# Supplementary material for: The NIRS Analysis Package: Noise Reduction and Statistical Inference
Source: PLoS One. 2011 Sep 2;6(9):e24322. doi: 10.1371/journal.pone.0024322 (PMC3166314; doi:10.1371/journal.pone.0024322)
Supplement: Appendix S2 — Single subject GLM analysis with FGLS. A detailed account of application of whitening using a power law covariance structure to offset autocorrelated errors in single subject inference of activation using a GLM. (DOC) [file pone.0024322.s004.doc]

## Appendix S2: single subject GLM analysis with FGLS

The most common method of analyzing functional imaging data is to carry out statistical inference using a general linear model (GLM - c.f. ), to single out which of the measured time series could be explained by the design of the experiment.

The difference between various models is the degree to which the shape of the HRF used in the model is constrained *a priori*, ranging from maximal constraints - i.e. using one canonical HRF for all participants and all brain regions – to minimal constraints – i.e. a finite impulse response (FIR) model that only assumes that responses are causal (occur after the stimulus) and are of a defined duration. The constraints placed on the shape of the HRF that the model allows are expressed in the choice of basis functions spanning the space of possible HRFs . The model vectors – taken as columns – are concatenated to form the design matrix *X*, and a column of ones is added to make the regression problem complete. If we denote the binary matrix indicating the occurrence of experimental events as *S* and the set of HRF basis functions as *hrf* we have:$X/1=S\otimes hrf$ where $X/1$ is the design matrix with the column of ones removed. If we denote a measured imaging time series (signal) by *y*, the design matrix of predictors as *X*, the unknown parameters as *b*, and the noise in the measurements (resulting both from the imaging system, and noise inherent to hemodynamics – e.g. systemic artifacts such as heart pulsation) as $\varepsilon$ , we obtain the following equation:

(1) ,

which is simply the matrix form of the following:

(2) $y(t)=b_{0}+b_{1}x_{1}(t)+...+b_{k}x_{k}(t)+\varepsilon(t)$.

Note that the columns of *X* need to be linearly independent for a unique solution to exist.

It is possible to add additional regressors to the design matrix, representing known confounds in the measured system (e.g. head movements in fMRI). In the approach we describe here, however, the known confounds for NIRS data are dealt with in the preprocessing stage of the analysis (see Methods section 2).

Once the system of equations in (1) is formed, it must be solved to estimate whether a time series contains a response to the experimental manipulation with respect to some degree of certainty. If we denote the solution to the equations by $\hat{b}$, then by (1), the estimate of the original signal would be $\hat{y}=X\hat{b}$. Similarly the estimate of the noise in the given experiment is denoted by *e*. Given that by definition the residuals are the difference between the original signal and the estimate, we can write $e=y-\hat{y}$, and $y=X\hat{b}+e$. The equations can be solved using the least squares method: i.e., minimizing . This insures an optimal solution, but only provided that the residuals are Gaussian and uncorrelated: i.e., $VAR(\varepsilon)=\sigma^{2}I$. In that scenario the explicit solution (referred to as the ordinary least square solution – OLS) is given by: $\hat{b}=(X^{t}X)^{-1}X^{t}y$.

If we denote $C=(X^{t}X)^{-1}$ , and , where *n* is the number of samples, k the number of coefficients, *H_0_*, *t_0_*, and *F_0_* refer to the null hypothesis and its *t* and *F* statistics respectively, and α is set as the desired probability of false positives, then the significance of the fit to the model can be assessed according to:

$H_{o}:t_{0}=0$ $H_{1}:t_{0}\neq0$ or,

$$H_{o}:Tb=0$$

$$H1:Tb\neq0$$

depending on the number of coefficients in the model (i.e., apply the *t* statistic if a single basis function is used for each condition and the *F* statistic otherwise), where *T* is a matrix of *r* independent contrast vectors. For example, in a two condition experiment modeled with a single basis function the corresponding contrast vector representing the hypothesis that the conditions differ in their magnitude of activation would be [0 1 -1].

The GLM framework can be adapted to accommodate autocorrelated noise by incorporating the correlation structure (the noise covariance matrix) into the model. We denote the noise covariance matrix $COV(\varepsilon)=\sigma^{2}V$. Since this is a covariance matrix, it is symmetric positive definite and therefore, has a square root (i.e., a matrix satisfying $V=KK$). As the covariance matrix is full-rank, it is invertible and hence this extends to the inverse as well, namely $V^{-1}=K^{-1}K^{-1}$. Therefore, by computing$K^{-1}$, we can define a new regression problem by multiplying the regression equation $y=Xb+\varepsilon$ by$K^{-1}$ on both sides, which writes$y^{*}=X^{*}b+\varepsilon^{*}$, where $y^{*}=K^{-1}y$ (the measured NIRS data multiplied by $K^{-1}$) and similarly for the noise $\varepsilon$ and the design matrix $X$. The first thing to notice is that solving this new problem does not change the estimate of the coefficients (we simply multiplied both sides of an equation with and invertible matrix). More importantly, if we compute the covariance of the residuals in the new problem we obtain:$COV(e^{*})=E(e^{*}e^{*})=E(K^{-1}ee^{T}K^{-T})=K^{-1}E(ee^{T})K^{-T}=K^{-1}\sigma^{2}VK^{-1}=\sigma^{2}I$. This newly defined regression problem therefore conforms to the assumptions of OLS; since this procedure results in uncorrelated residuals it is referred to as *whitening*. In fact, if we plug in $K^{-1}$ into to the equations in Methods section 2.3 we see that given *V,* the inference can be completed without computing $K^{-1}$ explicitly, i.e.:

$$\hat{b}=(X^{t}V^{-1}X)^{-1}X^{t}V^{-1}y$$

$$C=(X^{t}V^{-1}X)^{-1}$$

$$H_{o}:t_{0}=0$$

$$H_{1}:t_{0}\neq0$$

$$H_{o}:Tb=0$$

$$H1:Tb\neq0$$

Therefore, efficient and unbiased statistical inference can be carried out if the noise variance is known. In NIRS experiments this can be achieved by collecting rest data intermittently with evoked data. However, a more parsimonious option is to carry out feasible generalized least square analysis (FGLS). In FGLS, OLS is carried out first, followed by estimation of the noise covariance utilizing the residuals under the assumption that they conform to a presupposed structure. In the case of NIRS data, this is sound practice because the structure of the noise covariance in a given cortical locus is known up to a single parameter, namely the exponent of the power spectrum . We shall now describe the procedure of estimating the noise covariance matrix in more detail.

The autocorrelation of a time-varying process describes the correlation between values of the process at different points in time, as a function the time difference (lag): $R(\Delta t)=E\left[(x(t)-\mu_{t})(x(t+\Delta t)-\mu_{t+\Delta t})\right]/\sigma_{t}\sigma_{t+\Delta t}$. In the scenario of GLM analysis of NIRS data this simplifies to $R(t)=E\left[x(s)x(s+t)\right]/\sigma^{2}$. If we denote the power spectrum of a NIRS noise time series as $S(f)$ then according to the Wiener–Khinchin theorem$R(t)=\int_{-\infty}^{\infty} S(f)e^{2\pi ift}df$ or in the case of discrete measurements in terms of the discrete Fourier transform (DFT) $R(t)=\sum_{t=1}^{N} S(f)e^{2\pi it/N}=IDFT(S(f))$, where *t* is expressed in units of TR.

This theorem can be utilized to estimate the autocorrelation of noise the imaged cortical loci. To do so one needs to go through the following steps:

- *Carry out OLS regression and compute the residuals* $e=y-\hat{y}=y-X(X^{t}X)^{-1}X^{t}y$
- *Compute the power spectrum of the residuals* $S(f)=DFT(e)DFT^{*}(e)$

(* denotes the complex conjugate).

- *Regress the power spectrum to a line after transferring to log units:* regress log(*f*) to log(*S*(*f*)) to find the slope $\beta$
- *Compute the autocorrelation function:* i.e., $\hat{R}(t)=IDFT(f^{\beta})$
- *Build the covariance matrix.* Note that the covariance matrix is simply another way of arranging the autocorrelation function, namely: $COV(\varepsilon)_{ij}=\hat{R}(j-i)=Toeplitz(\hat{R}$
