# Supplementary material for: The NIRS Analysis Package: Noise Reduction and Statistical Inference
Source: PLoS One. 2011 Sep 2;6(9):e24322. doi: 10.1371/journal.pone.0024322 (PMC3166314; doi:10.1371/journal.pone.0024322)
Supplement: Appendix S3 — Group analysis using hierarchical GLMs with FGLS. A detailed account of group analysis applying a hierarchical GLM incorporating the single subject estimated error covariances to achieve optimal inference (through whitening). Method derived from [20]. (DOC) [file pone.0024322.s005.doc]

**Appendix S3: Group analysis using hierarchical GLMs with FGLS**

The following recapitulates and expresses their scheme in the present context. In the setting of imaging data analysis, group analysis is simply carrying out inference to see whether the effects examined at the single participant level are true at the group level. The group is treated as a random sample drawn from a population (random effects analysis). Just as the regression problem at the single participant level is expressed in terms of the parameters sought after (shape of HRF, level of activation, etc. …), so the hypotheses tested at the group level are expressed as hypotheses about samples of such parameters (e.g. the average level of activation, the average shape of HRF …). In this scenario the GLM is hierarchical because it expresses the hypotheses at both levels at the same time.

Each individual problem is denoted $y_{k}=Xb_{k}+\varepsilon_{k}$. This expresses the assumption that while the design is shared by all participants (and hence the design matrix has no index), both the noise (i.e. the associated covariance matrix) and the parameters (i.e. the HRF, or dynamics of the neuronal response for a given participant in a specific brain region in the experimental conditions) and hence the collected data are sampled randomly from a population distribution.

Accordingly, at the group level the model is (1) $y=Xb+\varepsilon$ (2) $b=X_{G}b_{G}+\eta$

where $y=\left[\begin{matrix} y_{1} \\ y_{2} \\ \vdots\\ y_{n} \end{matrix}\right]$, $X=\left[\begin{matrix} X & 0 & \cdots& 0 \\ 0 & X & & \vdots\\ \vdots& & \ddots& 0 \\ 0 & \cdots& 0 & X \end{matrix}\right]$, $b=\left[\begin{matrix} b_{1} \\ b_{2} \\ \vdots\\ b_{n} \end{matrix}\right]$, $\varepsilon=\left[\begin{matrix} \varepsilon_{1} \\ \varepsilon_{2} \\ \vdots\\ \varepsilon_{n} \end{matrix}\right]$

and $X_{G}$the group design$b_{G}$the vector of the group parameters (e.g. mean activation) and $\eta$ the group residuals. This can be expressed as a single regression problem i.e.: $y=XX_{G}b_{G}+\gamma$ where $\gamma=X\eta+\varepsilon$. If we denote the inner participant covariance as $COV(\varepsilon)=V$and the covariance at the group level (e.g. of activation, shape of the HRF…) as $COV(\eta)=V_{G}$ the associated covariance is $W=XV_{G}X^{t}+V.$

Therefore, the whitening (i.e., optimal) solution at the group level would be $\hat{b}_{G}=(X^{t}W^{-1}X)^{-1}X^{t}W^{-1}\hat{b}$. In our case, *V* would be given by extending the FGLS scheme from the single participant level to get:

$V=\left[\begin{matrix} (X^{t}V_{1}^{-1}X)^{-1} & 0 & \cdots& 0 \\ 0 & (X^{t}V_{2}^{-1}X)^{-1} & & \vdots\\ \vdots& & \ddots& 0 \\ 0 & \cdots& 0 & (X^{t}V_{n}^{-1}X)^{-1} \end{matrix}\right]$.

Given the usual sample sizes in imaging studies, it is necessary to employ a simple model for the group parameters namely to assume that the parameters are multi-Gaussian with the associated covariance matrix $V_{G}=\left[\begin{matrix} \sigma_{1}^{2} & 0 & \cdots& 0 \\ 0 & \sigma_{2}^{2} & & \vdots\\ \vdots& & \ddots& 0 \\ 0 & \cdots& 0 & \sigma_{k}^{2} \end{matrix}\right]$ or $diag(\sigma_{1}^{2}\cdots\sigma_{k}^{2})$. Thus the total covariance matrix would be:

$$W=\left[\begin{matrix} (X^{t}V_{1}^{-1}X)^{-1}+diag(\sigma_{1}^{2}\cdots\sigma_{k}^{2}) & 0 & \cdots& 0 \\ 0 & (X^{t}V_{n}^{-1}X)^{-1}+diag(\sigma_{1}^{2}\cdots\sigma_{k}^{2}) & & \vdots\\ \vdots& & \ddots& 0 \\ 0 & \cdots& 0 & (X^{t}V_{n}^{-1}X)^{-1}+diag(\sigma_{1}^{2}\cdots\sigma_{k}^{2}) \end{matrix}\right]$$

From this point onward estimation proceeds in the usual fashion, i.e.:

$$\hat{b}_{G}=(X^{t}W^{-1}X)^{-1}X^{t}W^{-1}\hat{b}$$

$$C=(X^{t}W^{-1}X)^{-1}$$

$$H_{o}:t_{0}=0$$

$$H_{1}:t_{0}\neq0$$

$$H_{o}:Tb=0$$

$$H1:Tb\neq0$$

Where *n* is the number of participants, *r* the number of independent contrasts and *k* the number of parameters in the model.
